# Enhancing Graphene Nanoplatelet Reactivity through Low-Temperature Plasma Modification

**DOI:** 10.1021/acsami.4c01226

**Published:** 2024-04-04

**Authors:** Karolina Kadela, Gabriela Grzybek, Andrzej Kotarba, Paweł Stelmachowski

**Affiliations:** Faculty of Chemistry, Jagiellonian University, Gronostajowa 2, 30-387 Krakow, Poland

**Keywords:** carbon materials, graphene, surface functionalization, low-temperature plasma, electron donor properties, postplasma reactivity, oxygen functional groups

## Abstract

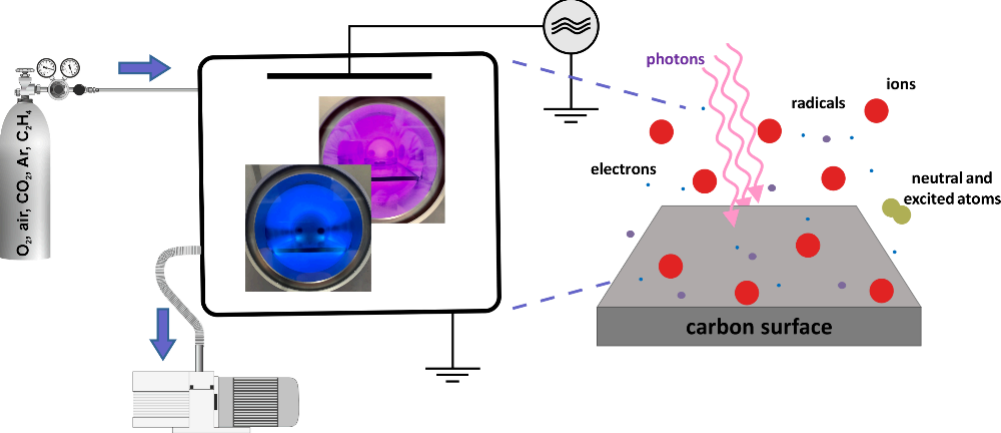

Graphene-based materials have great potential for applications in many fields, but their poor dispersion in polar solvents and chemical inertness require improvements. Low-temperature plasma allows the precise modification of materials, improving the physicochemical properties of the surface and thus creating the possibility of their potential use. Plasma treatment offers the possibility of introducing oxygen functional groups simply, rapidly, and in a controlled way. In this work, a systematic investigation of the effect of plasma modification on graphene nanoplatelets has been carried out to determine the optimal plasma parameters, especially the exposure time, for introducing the highest amount of oxygen functional groups on a surface. Different gases (O_2_, CO_2_, air, Ar, and C_2_H_4_) were used for this purpose. The chemical nature of the introduced oxygen-containing functionalities was characterized by X-ray photoelectron spectroscopy, and the structural properties of the materials were studied by Raman spectroscopy. The plasma-induced changes have been shown to evolve as the surface functionalities observed after plasma treatment are unstable. The immersion of the materials in liquids was carried out to check the reactivity of carbons in postplasma reactions. Stabilization of the material’s surface after plasma treatment using CH_3_COOH was the most effective for introducing oxygen functional groups.

## 1 Introduction

Graphene materials have exceptional electrical conductivity and flexibility and extended specific surface areas. Graphene flake is a light, transparent material with a specific capacitance of 1350 F g^–^^1^ and a large specific surface area of up to 2630 m^2^ g^–1^. This material has a porous structure and acidic and basic surface sites, greatly influencing its sorption capacity and catalytic properties. Graphene-type materials’ unique properties and structure make them suitable for developing various promising devices.^1^ However, although graphene-type materials possess impressive physicochemical properties, they often require surface functionalization to meet the needs of specific applications. The practical use of carbon powders in many fields needs to be improved by increasing their solubility in polar solvents and enhancing their affinity for most matrices. As a result, the usage of carbon materials is limited, and the main challenge is overcoming their hydrophobic and inert surface nature.^2^ Surface modification is essential to improve wettability and can be achieved through various processing techniques that modify their chemical composition and morphological properties.

One of the current challenges in developing carbon materials is finding ways to modify their surfaces precisely for the desired applications. There are two main methods of functionalizing carbon materials: noncovalent and covalent. Noncovalent functionalization involves substances interacting with the carbon material surface through van der Waals forces or π–π interactions. Covalent functionalization involves the permanent attachment of molecules of other substances to the surface of the carbon material, and the most common method of this type is surface oxidation.^3^ The presence of oxygen functional groups (OFG) stabilizes carbon materials’ dispersion in polar solvents and provides active sites for further modification. Surface functionalization also improves sorption properties and the deposition of the active phase for catalytic applications.

Modification of carbon materials by oxidation allows the introduction of various oxygen functional groups such as hydroxyl (−C–OH), ether or epoxy (−C–O–C−), carboxylic (−COOH), and carbonyl (−C=O) to the surface. Thermal oxidation is a convenient and universal method of obtaining carbon materials with a well-defined surface structure of oxygen functional groups. Although the amount of oxygen introduced to the carbon surface by thermal oxidation is lower than by oxidation with concentrated acidic solutions, the surface of the oxidized carbon remains uncontaminated with reaction byproducts as they are desorbed during the process. The resulting carbon preparation does not need to be subjected to the usually lengthy purification process and can be used for its intended purpose immediately after cooling, thus saving time.^4^ Harsh chemical oxidation with solutions of inorganic acids such as HNO_3_ or H_2_SO_4_ is the most common method for oxidizing the surfaces of carbon materials. The strong acid treatment effectively improves reactivity,^3^ and this type of functionalization allows a homogeneous material to be obtained. However, harsh methods involving high acid concentrations, long processing times, and high temperatures often cause structural damage and degradation of material properties. In addition, wet chemistry can be challenging to apply on an industrial scale due to toxic and hazardous elements as well as inconvenient handling and use of reagents. For these reasons, these techniques are not in line with the principles of “green chemistry”, and other methods are gaining interest.

Compared with the solution and thermal methods, plasma modification is becoming increasingly popular because of its many advantages. Low-temperature plasma is an efficient and universal technique for introducing various chemical moieties such as oxygen, amine, fluorine, or isocyanate onto the surface.^5−7^ It is a powerful technique for directly attaching oxygen-containing groups such as hydroxyl (−C–OH), ether or epoxy (−C–O–C−), carboxylic (−COOH), and carbonyl (−C=O) to a surface to improve reactivity. During plasma treatment, various effects occur, including surface cleaning and changes in the chemical composition of the surface, such as chemical functionalization and etching or amorphization.^8^ The low-temperature oxygen plasma treatment increases surface roughness and introduces oxygen functional groups into the graphene layer, weakening the sp^2^ hybridization of carbon and increasing the disorder of the material.^9−11^ These effects lead to an increase in the surface free energy and wettability of the material. In addition, mild conditions in terms of treatment time and temperature preserve the material’s bulk properties, i.e., it does not cause severe structural damage. Modification by plasma treatment has many advantages, such as a very short plasma treatment time to modify the material, environmental friendliness with low energy, and facile product recovery. Although the stability of the modification effects may be an issue, the application of direct postplasma treatment can stabilize the effects or even further modify the chemical composition of the surface.^12,13^ The studies indicate that air plasma allows the introduction of significant amounts of oxygen, but most of the reported research was conducted using oxygen plasma.^9,14^ We have recently shown that argon can create oxygen functional groups by activating the carbon surface and subsequent reaction with the oxygen from the ambient atmosphere.^12^ Additionally, plasma polymer films can be formed by providing an organic carbon source in the plasma system.^15^ Such an overlayer can also be a source of functional groups after in situ oxidation in the plasma chamber.

This work systematically studied the controlled functionalization of graphene nanoplatelets using low-temperature plasma and X-ray photoelectron spectroscopy. The study aimed to determine the plasma process conditions (power, pressure in the plasma chamber, treatment time) to introduce the maximum amount of oxygen functional groups (OFGs) for different gases applied without changing the material’s structure. Second, postplasma reactions with organic reagents to increase the number of OFGs were evaluated. Third, oxidation of the in situ formed plasma polymer film from C_2_H_4_ was assessed as a potential way to introduce oxygen functional groups.

## 2 Experimental Section

### 2.1 Materials

The raw graphene nanoplatelets (GNPs) were purchased from Nanografi Nano Technology, Germany. They had an average diameter of 30 μm, a surface area of 135 m^2^ g^–1^ with a thickness of 5 nm, and a conductivity in the range of 1.1 and 1.6 × 10^3^ S m^–1^.

### 2.2 Modification of GNPs by Low-Temperature Plasma

The surface functionalization was performed using a commercial cold plasma system (Femto-Diener electronic GmbH, Nagold, Germany) with a low-frequency generator of 40 kHz. The geometry of the plasma chamber is round with quartz glass walls. The inner diameter of the chamber is 95 mm and the depth is 320 mm (approximate volume is 2 L. Maximum power of the generator is 100 W; however, a linear response to the setting with the manual knob starts from about 30 W. The electrical scheme of the device is depicted in Figure S1. The flow rate of oxygen resulting in 0.2 mbar pressure in the chamber is 5 sccm, and for 0.8 mbar, it is 39 sccm. 5–10 mg of the powder material was placed onto the quartz tray in the middle of the plasma chamber. Different gases were used for the studies: oxygen (Air Products, 99.9998% O_2_), air (Air Products, X40S COM, 2.2), Ar (Siad, 99.9998%), CO_2_ (Air Products, X50S 37.5 K, ultrapure), and C_2_H_4_ (Siad, 99.9998%). The low-temperature plasma chamber of the apparatus was connected to a suitable gas source, an electric field generator, and a vacuum pump. Graphene nanoplatelets were treated with plasma and tested as soon as possible after modification of the material (ASAP) and after contact with water or CH_3_COOH and water and drying at 60 °C via lyophilization.

A reference sample of graphene nanoplatelets was modified by an acidic solution method. The material was placed in a round-bottom flask and flooded with 1 M ammonium persulfate (APS) solution in 2 M H_2_SO_4_. The mixture was heated at 60 °C for 24 h. The material was then centrifuged, rinsed several times with distilled water, and dried at 60 °C. The description of the adopted coding of the samples is collected in Table 1. For example, air + CH_3_COOH means that the sample has been air plasma-modified GNPs reacted with CH_3_COOH and rinsed with H_2_O, 5:1 C_2_H_4_:CO_2_ + H_2_O means that the sample has been ethylene and carbon dioxide plasma-treated GNPs with a 5:1 ratio of gases reacted with H_2_O.

**Table 1:** Description of Samples’ Coding Used in the Manuscript

sample coding element	description
ref	unmodified GNPs
ref + APS	ammonium persulfate modified GNPs
ASAP	the measurement taken as soon as possible after plasma treatment
air, O_2_, Ar	air, oxygen, argon plasma-modified GNPs
X:Y C_2_H_4_:CO_2_	ethylene + carbon dioxide plasma with a specified ratio of gases
H_2_O	the measurement taken after plasma treatment and reaction with H_2_O
CH_3_COOH	the measurement taken after plasma treatment and reaction with CH_3_COOH and rinsing with H_2_O

### 2.3 Work Function Measurements

The work function (ϕ, WF) changes of carbon materials samples were determined based on contact potential difference (*V*_CPD_) measurements by the dynamic condenser of the Kelvin method with a KP6500 probe (McAllister Technical Services). The reference electrode ϕ_ref_ made from a stainless steel plate with a 3 mm diameter and reference work function ϕ_ref_ ≈ 4.3 eV. The measurements were performed with a vibrational frequency of 114 Hz, amplitude of 40 au, and a peak-to-peak gradient versus backing potential of 0.1. The five backing potentials were applied to get a single value of contact potential difference (CPD); the one value was an average of 32 independent measurements. The work function value changes due to the plasma treatment were equal to the differences in the CPDs before and after the plasma modification. The value of the work function was calculated based on the eq 1.
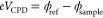
1

### 2.4 X-ray Photoelectron Spectroscopy

X-ray photoelectron spectroscopy (XPS) was used to investigate the reference and modified powder GNP samples using a SESR4000 analyzer (Gammadata Scienta) in a vacuum with a base pressure below 5 × 10^–9^ mbar. A monochromatic Al–Kα source with 250 W at 1486.6 eV emission energy was used, and the pass energy for selected narrow binding energy scans was 100 eV. The penetration depth is typical for XPS measurements and equals 1–10 nm. CasaXPS version 2.3.24PR1.0 was used to process the raw data.^16^ The binding energy scales were corrected for the gold work function determined in the spectrometer, 4.65 eV.

The analysis of the XPS data is central to this study. The detailed fitting procedure is presented here to ensure curve fitting of the C 1s spectral range is as objective as possible.^17^ First, the reference GNP C 1s spectrum was fitted with a minimal number of components, considering their metallic properties, presence of defects, and shake up π–π* resonance band.^18^ This set of bands was further used as a basis for the curve fitting of surface-modified graphene nanoplatelets. The formed oxygen groups were modeled with three bands representing the general chemistry of surface oxygen groups, namely COO-type, C=O-type, and C–O-type groups.^19^ Care was taken to ensure that these bands’ position and full width at half-maximum (fwhm) values were within the reported limits. The fitted spectra were checked for consistency within the studied series of surface modifications.

### 2.5 Raman Spectroscopy

The micro-Raman spectra were acquired by using a Renishaw InVia spectrometer coupled to a Leica DMLM confocal microscope. The excitation wavelength was 514.5 nm, and the objective magnification was 50x. Ten scans were taken for each sample with a resolution of 1 cm^–1^ to obtain sufficient signal-to-noise. The spectra were collected in the spectral range of 1000–3000 cm^–1^.

### 2.6 Temperature-Programmed Desorption with Quadrupole Mass Spectrometric Detection

For the temperature-programmed desorption (TPD) measurements, 50 mg of sample was used. The sample was stabilized for 30 min at a 20 mL min^–1^ He flow rate at 30 °C. Then, at a flow rate of 20 mL min^–1^ He, thermodesorption was carried out with a temperature rise of 10° min^–1^. The evolved gas composition was monitored with a Prevac mass spectrometer (*m*/*z* = 4, 12, 16, 17, 18, 28, 29, 32, 43, 44).

## 3 Results and Discussion

### 3.1 Stability of Tested Materials

**Figure 1:**
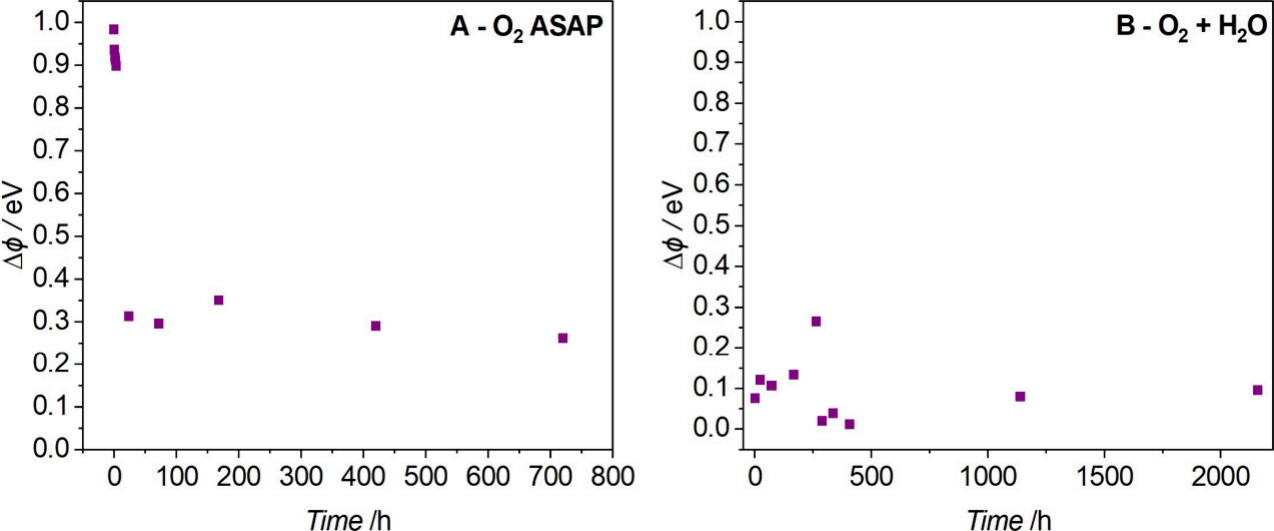
Work function changes of GNPs modified by oxygen plasma: (A) just after plasma modification (ASAP) and (B) after contact with water.

First, the stability tests of the plasma-treated GNPs were carried out using a Kelvin probe to follow the changes in surface properties. For this purpose, graphene nanoplatelets were modified using oxygen plasma parameters: time of 6 s, power of 100 W, and pressure of 0.2 mbar. The work function changes were measured as soon as possible after the plasma treatment. The same plasma treatment was applied to another GNP sample subjected to water and dried immediately after the modification. Figure 1 shows the stability of the materials modified by oxygen plasma (time 6 s, power 100 W, and pressure 0.2 mbar).

Plasma enables the functionalization of a material by introducing polar functional groups onto its surfaces, forming surface dipoles and increasing the carbon’s material work function.^20,21^ The experimental work function value of the untreated graphene surface was ∼4.4 eV. However, after plasma treatment, the work function increased to 5.4 eV (1 eV difference). Duch et al. found that the more powerful plasma conditions were applied, the higher work function changes were observed.^22^ The plasma conditions refer to a higher plasma generator power, longer plasma treatment time, and lower oxygen partial pressure. The effect of modification of plasma-treated carbon materials is temporary and decays exponentially just after the plasma treatment due to electrostatic charging and the recombination of oxygen functional groups.^12,23^ After the plasma-treated sample was contacted with water, the work function values were stable and higher than the reference value; however, they were much lower than values measured ASAP just after plasma treatment, as presented in Figure 1.

### 3.2 Plasma Treatment with O_2_, Air, and Ar Gases

A series of XPS studies using three different oxygen plasma conditions were carried out (Table 2). According to the fitting procedure described in Section 2, the XPS C 1s spectra revealed the presence of the main graphitic carbon asymmetric peak at 284.4 eV (sp^2^ carbon in C=C) and components due to defects around 284 eV, disordered structure around 285.3 eV, and π–π* shakeup feature above 290 eV. Furthermore, three generic oxygen surface groups were determined with peaks at 285.9–286.6 eV associated with single bonded oxygen (C–O), at 286.7–287.5 eV due to double bonded oxygen (C=O), and at 288.3–288.9 eV carboxyl or ester type group (O–C=O). For each type of modification, the measurements were performed immediately after plasma treatment (ASAP), after immersing the modified material in water or acetic acid and rinsing in water, then using a lyophilizer and drying at 60 °C. The optimization of the drying procedure is described in the Supporting Information (Table S1 and Figure S2). The conditions that yielded the highest surface oxidation degree were also used for air and argon plasma treatment. The results obtained previously for graphene paper signify that immersion of the carbon material in water allows obtaining a stable surface, demonstrated by the constant value of the work function, but also by increasing the concentration of OFGs on the surface compared to the sample treated with plasma only.^12^ Based on these results, graphene nanoplatelets were similarly immersed in water or acetic acid and rinsed with water to study postplasma reactions. The XPS spectra were also taken for the reference GNPs without plasma modification but with the same postplasma treatments, that is, (1) after wetting with water and drying and (2) after wetting with acetic acid, rinsing with water, and drying. The reference wet oxidation was performed with the APS (Table 2 and Figure S3).

**Table 2:** XPS-Derived Total Surface Oxygen Content and Relative Amount of Oxygen Functional Groups for Modified Graphene Nanoplatelets by Various Types of Gases under Different Conditions

	element content/at.%			type of functional group			
	%O	%N	%C	CO	C=O	COO	∑
reference	5	1	94	4.7			4.7
reference + H_2_O	4.7	0.9	94.4	4.6			4.6
reference + CH_3_COOH	5.2	0.8	94	5.3			5.3
reference + APS^a^	12.8	1.8	83.8	3.5	3.1	3.1	12.8
O_2_ (1 min, 0.2 mbar)							
ASAP	9.3	0.7	90	3.4	3	1.5	9.4
+H_2_O	7.4	0.4	92.2	2.1	3.3	1.2	7.8
+CH_3_COOH	7.6	1	91.5	2.4	2.4	1.4	7.6
O_2_ (5 min, 0.7 mbar)							
ASAP	9.3	0.8	90	3	2.8	2	9.9
+H_2_O	9.2	1	89.8	2.4	3.3	2	9.6
+CH_3_COOH	10.7	0.9	88.4	5.8	2.9	1.2	11.1
O_2_ (5 min, 0.2 mbar)							
ASAP	10.6		89.4	4.4	4.4	2.2	11
+H_2_O	6.8	0.2	93	2.9	2.6	0.5	6.4
air (5 min, 0.7 mbar)							
ASAP	13.1	0.4	86.6	5.4	3.7	1.6	12.3
+H_2_O	7	0.9	92.2	2.8	2	0.9	6.6
+CH_3_COOH^b^	13.1	1.3	85.6	7.2	3.1	1.6	13.5
Ar (5 min, 0.7 mbar)							
ASAP	8.1	0.7	91.2	3.5	2.3	1.1	8
+H_2_O	5.9	1	93.1	2.6	1.6	0.7	5.6
+CH_3_COOH	10.9	0.7	88.4	4.8	3.8	1.1	10.8

a Graphene nanoplatelets modified by APS (wet method).

b Sample used for the TPD studies.

**Figure 2:**
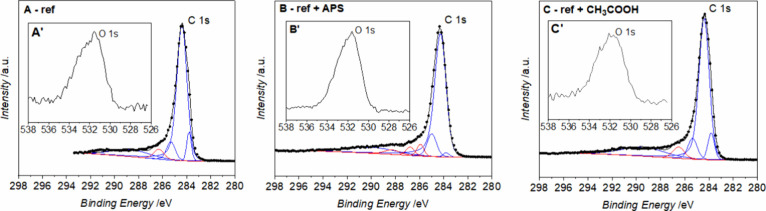
XPS C 1s and O 1s (insets A’, B’, C’) spectra and curve fitting of graphene nanoplatelets, respectively, for (A) reference, (B) after APS treatment, and (C) sample treated with the air plasma (5 min, 0.7 mbar) and reacted with CH_3_COOH.

The reference material of unmodified graphene nanoplatelets immersed in water and acetic acid indicates an oxygen content of around 5%, comparable to the dry, initial GNP powder. Graphene nanoplatelets modified with APS as an example of a wet method allow for the introduction of as much as 12.8% O. Analogous effect of implementation OFGs onto the surface is possible modifying the surface with using air plasma under conditions 5 min and 0.7 mbar and then immersed in CH_3_COOH and rinsed H_2_O, afterward lyophilized and dried at 60 °C. The examples of the curve fitting are presented in Figure 2.

**Figure 3:**
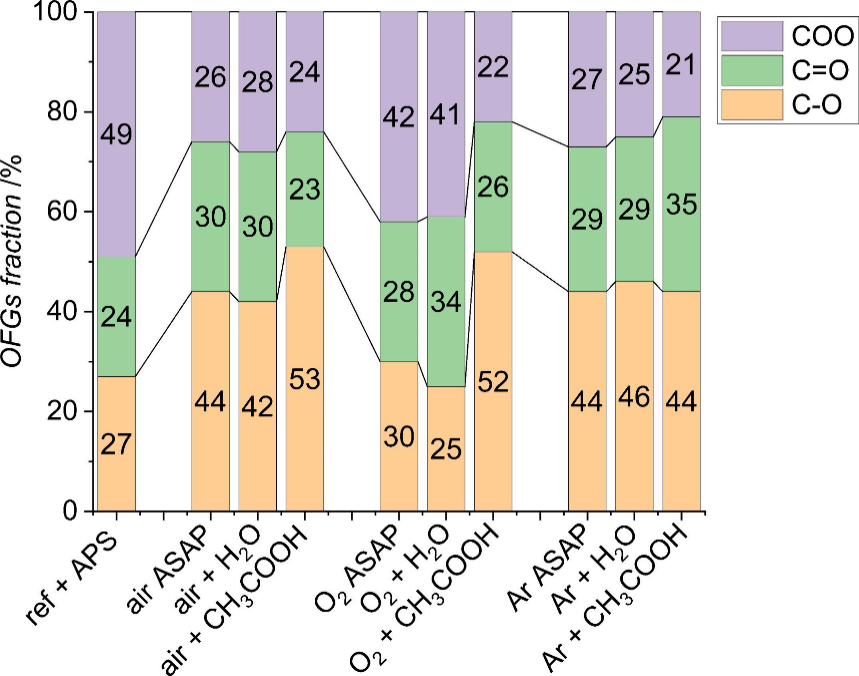
Distribution of various oxygen functional groups introduced by plasma modification and postplasma reactions on the surface of graphene nanoplatelets and the reference APS-modified sample.

The quantitative analysis based on the O 1s and C 1s spectral ranges yielded the highest surface oxygen content for the air plasma-modified GNPs, similar to the APS oxidized sample. Thus, the C 1s spectra with the fitted peaks of C 1s components are presented in Figure 3 for these samples. All recorded spectra and fitted components are collected in the Supporting Information (Figures S3–S6).

The most desirable effect among the tested gases is plasma after treatment with acetic acid, which correlates with the previous findings for the graphene paper (Graphene Laboratories, Calverton NY, USA, thickness 25 μm, density 2 g cm^–3^, conductivity 3.19 × 10^5^ S m^–1^).^12^ However, comparing the GNP and graphene paper materials treated similarly with oxygen, air, and argon plasma, it can be concluded that significantly less surface oxygen is incorporated on the surface of the powder material of graphene nanoplatelets than for the graphene paper. In the next stage, the distribution of oxygen functional groups introduced to the surface as a result of various modifications was examined, and the results are presented in Figure 3.

Recent studies conducted for mesoporous carbon materials to investigate the role of speciation of oxygen functional groups in the oxygen evolution reaction have shown that the significant effect on surface functionalization is not the total amount of oxygen introduced to the surface but the speciation of the functional groups. The carboxylic oxygen groups were found to control the dispersion and activity of the catalyst.^24^ Therefore, the performed functionalization of GNPs can be analyzed regarding the relative abundance of COO-type surface groups. The modification made with APS was the most effective because almost 50% of the functional groups are COO groups. Oxygen plasma also introduces many COO-type groups, but acetic acid reduces their amount. Taking into account the set of samples modified with an inert gas—argon, it can be concluded that despite the different total amounts of oxygen introduced to the surfaces, 8, 6, and 11%, respectively, the distribution of hydroxyl, carbonyl, and carboxyl groups remains practically unchanged at 45, 31, and 24%.

### 3.3 Plasma Polymer Film Formation and Oxidation

**Figure 4:**
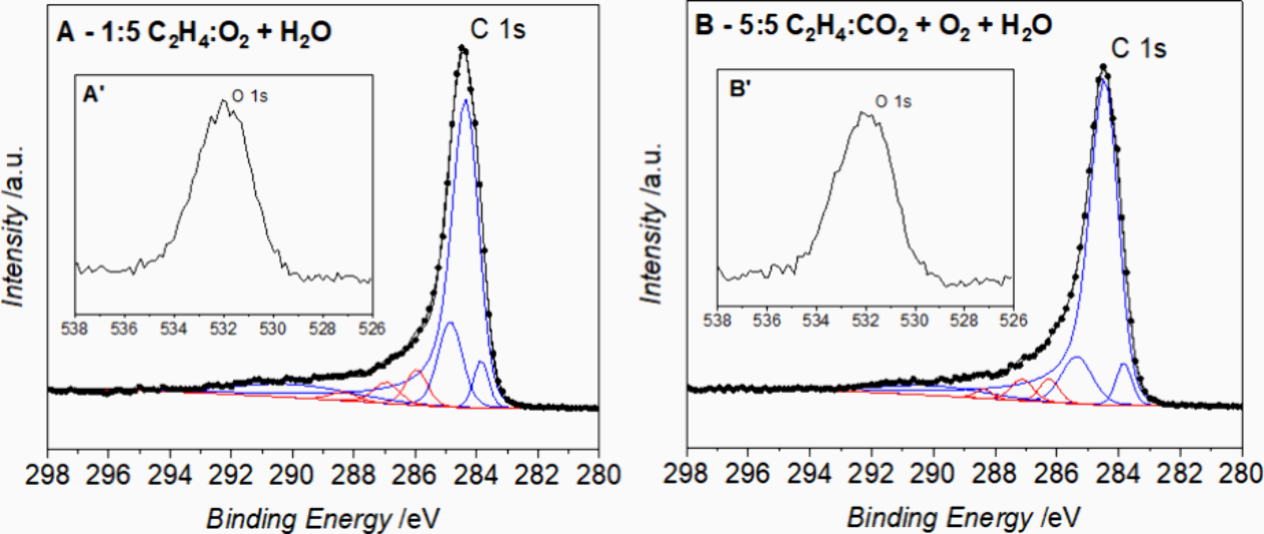
XPS C 1s and O 1s (inset) spectra and curve fitting of graphene nanoplatelets treated with ethylene plasma with (A) oxygen and (B) carbon dioxide and subsequent oxygen plasma.

A series of tests were performed to thoroughly investigate the effect of the polymer-forming and oxidizing gas on graphene nanoplatelets (Table 3 and Figure 4). C_2_H_4_ was used to form a plasma polymer layer, and CO_2_ and O_2_ were used as oxidizing gases to oxidize the resulting modified surface in situ. A mixture of C_2_H_4_:CO_2_ in a 5:5 ratio was used for modification, which, based on the previously reported results for graphene paper, indicated the most effective modification.^12^ Without removing the material from the plasma, it was treated with oxygen plasma at 0.2 mbar, and time optimization was performed (6 s–5 min), and the results are presented in Figure S7. Additional measurements were also performed for samples postplasma treated with acetic acid and changing the time or pressure and the oxidant to air, as shown in Table 3.

**Table 3:** XPS Total Content of Elements on the Surface for Modified Graphene Nanoplatelets and Optimization of Process Conditions Using the Mixture of C_2_H_4_:CO_2_

	element content/at.%		
sample	%O	%N	%C
5:5 C_2_H_4_:CO_2_ (5 min, 0.8 mbar) + optimization of time for O_2_ (0.2 mbar) + H_2_O			
6 s	7.9	0.7	91.4
30 s	10.8	0.9	88.3
60 s^a^	10.9	0.5	88.6
5 min	8.8	0.8	90.4
5:5 C_2_H_4_:CO_2_ (5 min, 0.8 mbar) + O_2_ 5 min, 0.7 mbar			
H_2_O	8	0.3	91.7
CH_3_COOH	7.8	0.7	91.5
5:5 C_2_H_4_:CO_2_ (5 min, 0.8 mbar) + O_2_ 1 min, 0.2 mbar			
CH_3_COOH	8	0.5	91.5
5:5 C_2_H_4_:CO_2_ (5 min, 0.8 mbar) + air 5 min, 0.7 mbar			
H_2_O	8.3	0.4	91.3
CH_3_COOH	8.3	0.2	91.5

a Sample used for the TPD studies.

The results indicate that for a 5:5 C_2_H_4_:CO_2_ ratio with 5 min and 0.8 mbar and a further 30 or 60 s treating the material with O_2_ introduces the highest amount of surface oxygen (∼11%). However, the XPS spectra of these samples show that this modification renders the material nonconductive. It is manifested by a double maxima in the C 1s and O 1s bands due to energy shift between conducting and nonconducting parts of the surface (Figure S7). The overlapping double maxima in the C 1s and O 1s bands make curve fitting impossible, and thus, the speciation of the surface oxygen groups cannot be determined. To verify the hypothesis of possible partial mild oxidation of the polymeric layer, an attempt was made to optimize the oxidant in the C_2_H_4_:CO_2_ ratio in the gas mixture at 5 min and 0.8 mbar or 0.2 mbar (Table 4 and Figure S8). The optimization of the ratio in the mixture C_2_H_4_:O_2_ under plasma conditions 5 min, 0.8 mbar was also performed, as shown in Figure S9.

**Table 4:** XPS Total Content of Elements on the Surface for Modified Graphene Nanoplatelets and Optimization of the Ratio Oxidant (CO_2_ or O_2_) in the Mixture C_2_H_4_:CO_2_ or C_2_H_4_:O_2_

	element content/at.%		
plasma composition and sample treatment	%O	%N	%C
C_2_H_4_:CO_2_ (5 min, 0.8 mbar) + H_2_O			
1:05	6.3	0.9	92.8
2:05	6.8	1.2	92
3:05	6.7	0.6	92.6
4:05	5.9	0.4	93.7
5:05	5.6	1.5	92.9
C_2_H_4_:CO_2_ (5 min, 0.2mbar) + H_2_O			
5:05	6.9	0.4	92.7
C_2_H_4_:O_2_ (5 min, 0.8 mbar) + H_2_O			
1:01	6.8		93.2
5:01	6.1	1.4	92.6
1:5^a^	10.1	2	87.9

a Sample used for the TPD studies.

The total amount of surface oxygen in samples after the in situ oxidation with CO_2_ varies between 5.6 and 6.9% as presented in Table 4. This number is relatively low because the reference material contains 5% surface oxygen. The highest amount of introduced oxygen (10%) was obtained with oxygen as an oxidant molecule using C_2_H_4_:O_2_ with a 1:5 ratio. However, this effect can be mainly related to the oxygen plasma cleaning the surface and oxidizing it because the results are similar to those given in Table 2 for the modification of graphene nanoplatelets by oxygen plasma in 0.7 mbar and 5 min.

### 3.4 Thermal Stability of Functionalized Graphene Nanoplatelets

**Figure 5:**
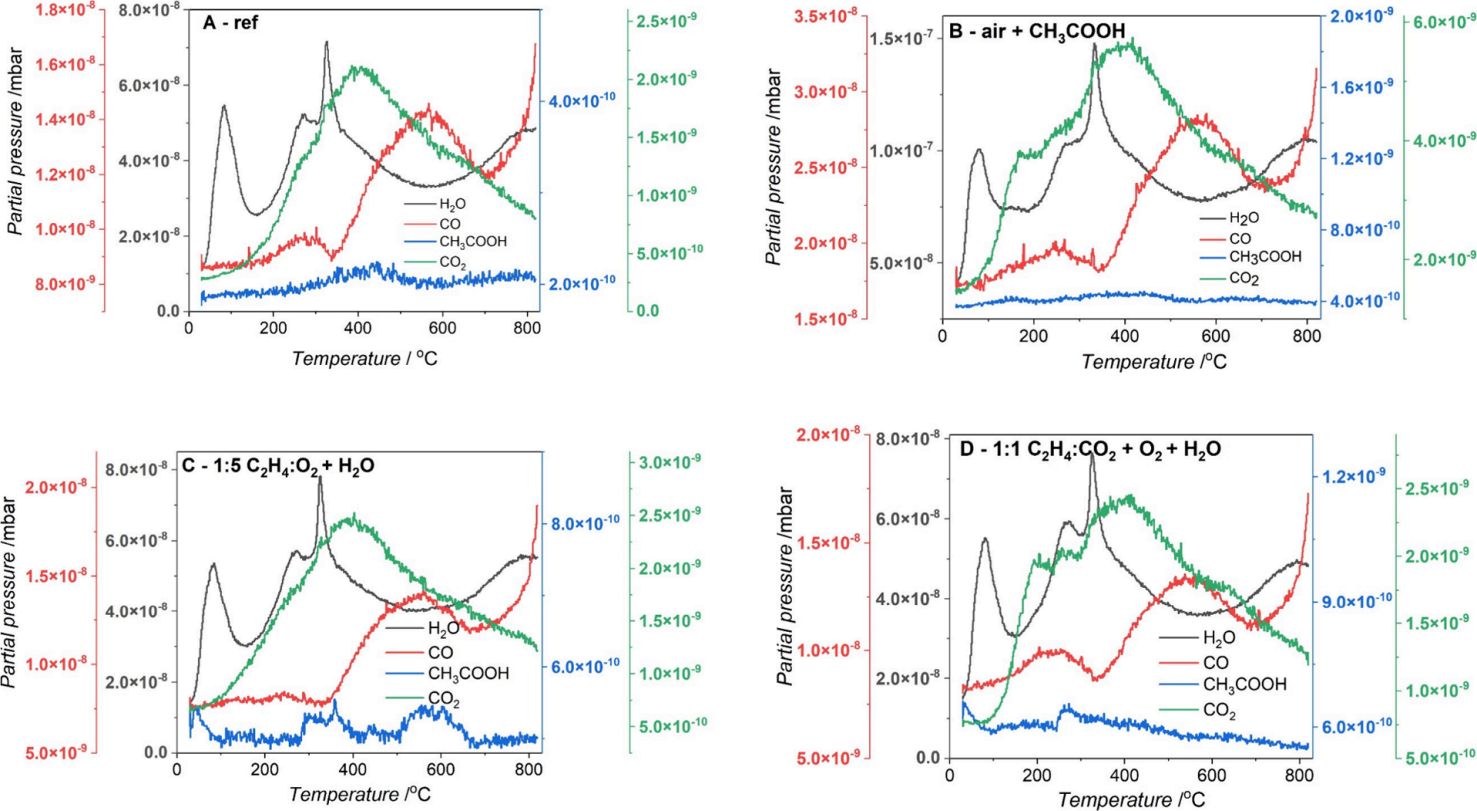
TPD experimental profile for the graphene nanoplatelets (A) unmodified, (B) modified by air plasma under plasma conditions 5 min, 0.7 mbar pressure, and acetic acid, (C) treated with 1:5 C_2_H_4_:CO_2_ under plasma conditions 5 min, 0.8 mbar pressure, (D) treated with 1:1 C_2_H_4_:CO_2_ under plasma conditions 5 min, 0.8 mbar pressure and O_2_ with 1 min and 0.2 mbar pressure.

TPD analysis was performed for the reference GNPs and selected plasma-modified samples. In the TPD experiments, the main desorbing gases are water, carbon monoxide, and carbon dioxide. CH_3_COOH was also included in the measurements as it was used for the postplasma surface modification. Figure 5 shows the results of the H_2_O, CO_2_, and CO and CH_3_COOH desorption experiments on the selected samples.

**Figure 6:**
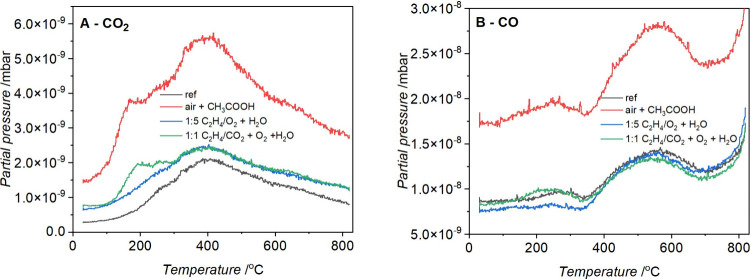
Experimental TPD profiles of (A) CO_2_ and (B) CO from modified graphene nanoplatelets.

Although the samples’ TPD profiles have a similar general shape, they differ in intensity, possibly due to variations in the spectrometer’s background or the sample amount. The plasma-modified sample released significantly more H_2_O than the reference, possibly due to its adsorption on functional groups, which macroscopically increased the surfaces’ hydrophilicity.^25^ To directly compare the CO_2_ and CO desorption from the studied samples, Figure 6 displays collected TPD profiles.

In Figure 6A, each tested material demonstrates a prominent CO_2_ peak around 400 °C accompanied by shoulders at approximately 250 and 600 °C. Notably, both the air plasma and 1:1 C_2_H_4_:CO_2_ plasma-modified materials reveal an additional CO_2_-TPD peak at about 180 °C, indicating the presence of new surface functional groups binding H_2_O. This peak is absent for reference and 1:5 C_2_H_4_:CO_2_ plasma-treated samples. The literature data suggests that the CO_2_ peak at lower temperatures can be attributed to a carboxylic functional group, while the peak at temperatures higher than 400 °C can be assigned to lactone groups.^26^ All studied materials showed CO desorption, usually attributed to the decomposition of ethers and carbonyls at temperatures 500 °C or higher.^27^ It is worth mentioning that the CO signal also comes partly from the CO_2_ fragmentation. The desorption of CH_3_COOH, which was used for the air-plasma-treated sample, was also followed, indicating the possible presence of acetic acid residues. The desorption peaks are relatively broad and overlapping, and the interpretation of TPD spectra in the literature is ambiguous.^28^ Therefore, additional insight into the surface properties from the thermal stability studies is limited.

### 3.5 Raman Spectroscopic Studies

**Figure 7:**
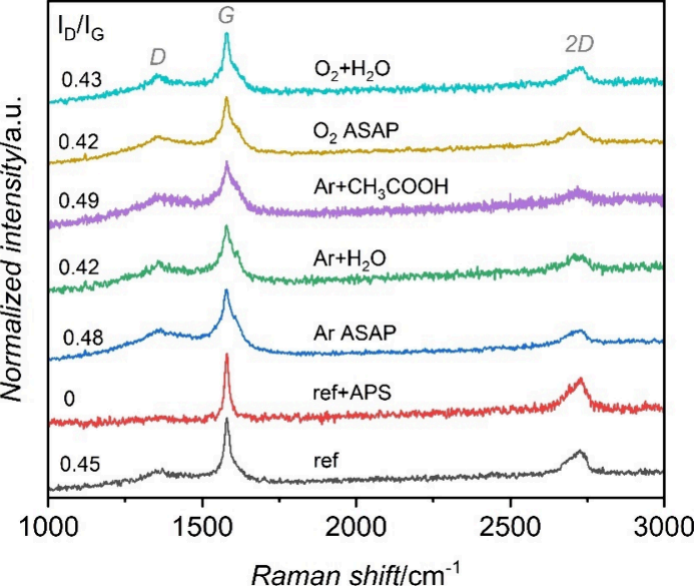
Raman spectra together with the corresponding *I*_D_/*I*_G_ ratios for reference GNPs and samples modified by APS and plasma treatment.

To verify the effect of the surface functionalization with low-temperature plasma on the structure of graphene nanoplatelets, the spectroscopic Raman characterization was performed. The Raman spectrum for reference GNPs is compared in Figure 7 with that of the APS-modified sample and the representative spectra of samples treated with argon and oxygen plasma. In all cases, two intense bands distinctive for graphene structure, G (∼1578 cm^–1^) and 2D (∼2730 cm^–1^), are present. The G band relates to the stretching motion of ordered sp^2^ bonds between carbon atoms, while the 2D band relates to the two-phonon lattice vibrational process. The observed 2D band shape, consisting of several components, is typical of multilayer graphite materials, as well as bulk graphite. In all spectra, except the one for the APS-treated sample, a very low-intensity peak appears at ∼1353 cm^–1^ (D-band), associated with the presence of a small fraction of disordered carbon. Similarly, the D’ band at ∼1620 cm^–1^, which is associated with the carbon amorphization process, observed for most of the samples, is absent for the APS-treated sample.^12^

Regardless of the gas used for plasma treatment, no significant changes in the Raman spectra upon plasma modification are observed. For each of the gases employed during the plasma functionalization of GNPs, the *I*_D_/*I*_G_ ratios are almost unchanged with respect to the reference sample. This indicates no significant influence on the graphene structure of the GNPs. In turn, a significant change in the structure of the GNPs is observed for APS treatment, which removed a part of the disordered carbon structures from the GNPs sample.

## 4 Summary and Conclusions

Graphene-based materials have great potential but very often require modification to be used in a specific application. Low-temperature plasma treatment is a promising method for precisely functionalizing carbon surfaces by introducing oxygen functional groups to improve their physicochemical properties. Systematic research on the functionalization of graphene nanoplatelet surfaces with the use of plasma was carried out in this work.

Since surface changes induced by plasma treatment are unstable, measurements were performed immediately after modification and after the modified material was immersed in water or acetic acid. Regardless of the type of gas used and the time of exposure to plasma treatment, the Raman spectra do not indicate significant changes in the bulk structure of the tested material, as evidenced by the nearly constant values of the calculated *I*_G_/*I*_D_ ratios. The use of argon plasma yielded substantial surface functionalization, indicating the reaction of the plasma-activated surface with the oxygen after its exposure to the ambient atmosphere. Even though the total amount of surface oxygen differed in the argon-modified samples, the distribution of oxygen functional groups remained almost unchanged.

Graphene nanoplatelets modified with air plasma, immersed in CH_3_COOH, and rinsed with H_2_O allowed for introducing a comparable amount of OFGs to the surface as in the case of modification with APS*,* around 12 at. % oxygen. However, APS modification was the most effective for the desired oxygen speciation, with almost 50% of the functional groups being carboxylic-type groups. For that effect, oxygen plasma also introduced a significant amount of these groups, approximately 40%. However, APS treatment involves concentrated reagents, long processing times, and high temperatures, making it a time-consuming method with a lot of waste to dispose of. Therefore, the plasma-based method can be used quickly and more effectively to improve carbon materials’ reactivity for further applications. XPS spectra fitting was performed to identify which modifications allowed for the introduction of the most significant number of carboxylic surface groups.

Much of the research focused on the oxidation of the in situ formed plasma polymer film made with the organic precursor—this functionalization results in a surface layer that is reactive and rich in radicals. The hypothesis was that in situ oxidation of the polymer layer can lead to an increased concentration of oxygen functional groups. However, the obtained XPS spectra suggest that the resulting material is partially poorly conductive when a high ratio of C_2_H_4_ to oxidizing gas is used, indicating limited film oxidation. A consecutive plasma polymer film formation and in situ oxidation with oxygen in the second step led to surface functionalization similar to using only oxygen plasma treatment. These negative results indicate the limited applicability of this approach.

Plasma modifications have shown great potential for introducing significant amounts of oxygen functional groups on the surface materials for various gases without altering the bulk structure. Postplasma reactions suggest that the quantity of these groups can be increased, particularly with the use of acetic acid, leading to improved reactivity and expanded possibilities for future applications. It has been confirmed that oxidizing in situ formed plasma polymer layers can introduce oxygen functional groups. However, their number is much lower than that for argon or oxidative plasma treatments.

## Supporting Information Available

Additional experimental details of the plasma setup and C 1s and O 1s X-ray photoelectron spectra of every sample reported in this study (PDF)
