# Supplementary material for: Enhancing Graphene Nanoplatelet Reactivity through Low-Temperature Plasma Modification
Source: ACS Appl Mater Interfaces. 2024 Apr 4;16(15):19771–9. doi: 10.1021/acsami.4c01226 (PMC11040526; doi:10.1021/acsami.4c01226)
Supplement: Supplementary file 1 — am4c01226_si_001.pdf [file am4c01226_si_001.pdf]

Supporting Information

for

Enhancing graphene nanoplatelet reactivity through low-temperature plasma modification

Karolina Kadela, Gabriela Grzybek, Andrzej Kotarba, Paweł Stelmachowski*

Jagiellonian University, Faculty of Chemistry, Gronostajowa 2, 30-387 Krakow, Poland

* pawel.stelmachowski@uj.edu.pl

The electrical scheme of the plasma device.

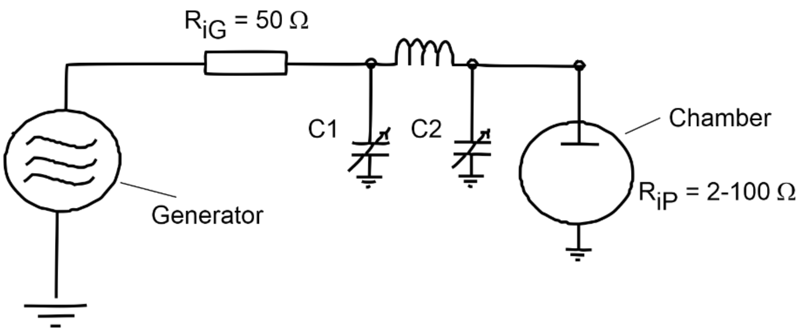

Figure S1. A scheme of the plasma device.

Optimisation of the drying procedure

Two measurements were conducted to compare the effectiveness of different drying conditions. Modifications of graphene nanoplatelets were performed with the same plasma parameters 5 min, 0.7 mbar, 100 W using oxygen as the feed gas and then immersed in CH_3COOH and rinsed out several times. In the first case, the modified material was lyophilised and dried at 60°C for 24 h. In the second case, the sample was dried in a vacuum dryer. The outcomes achieved are shown in Table S1, and appropriate XPS spectra are presented in Figure S1.

Table S1. XPS-derived total surface oxygen content and relative amount of oxygen functional groups for graphene nanoplatelets dried in different conditions.

	Element content/at.%			Type of functional group			
	**O**	**N**	**C**	**CO**	**C=O**	**COO**	$\sum$
O_2 (5 min, 0.7 mbar)							
$+CH_3COOH$	10.7	0.9	88.4	6.9	3.0	0.6	11.1
$+CH_3COOH$+VAC	7.4	0.9	91.7	3.0	2.2	1.0	7.2

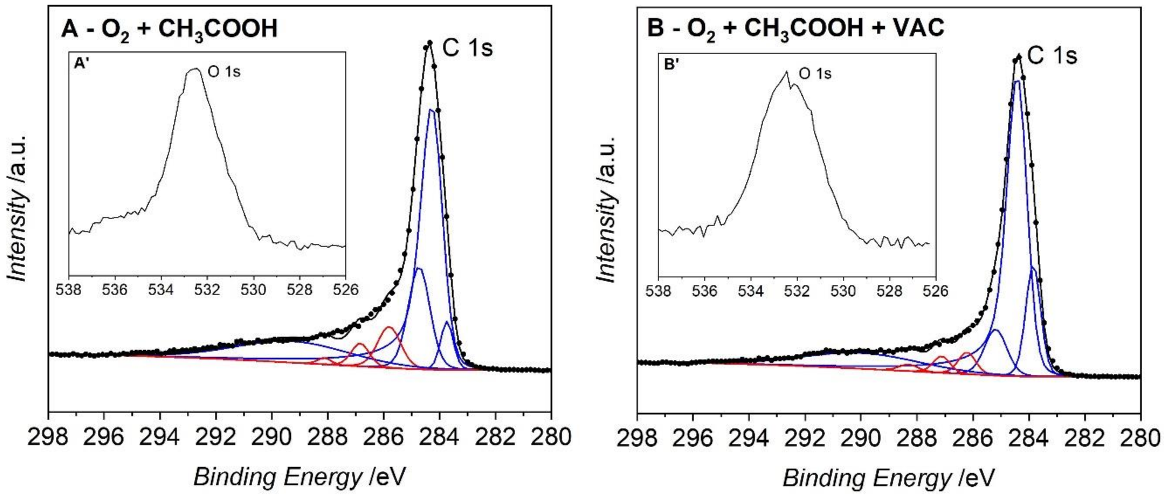

Figure S2 XPS O 1s and C 1s and deconvolution of graphene nanoplatelets dried in different conditions: A – lyophilised and dried at 60 °C, B – vacuum dried.

The material subjected to drying by lyophilisation shows a greater than 3% total oxygen content in the sample compared to the vacuum dryer. Figure 23 presents the variable distribution of functional groups attached to carbon. Therefore, all measurements were made by lyophilisation and drying at 60 ^{o}C.

XPS spectra and numerical data summary for the modified graphene nanoplatelets

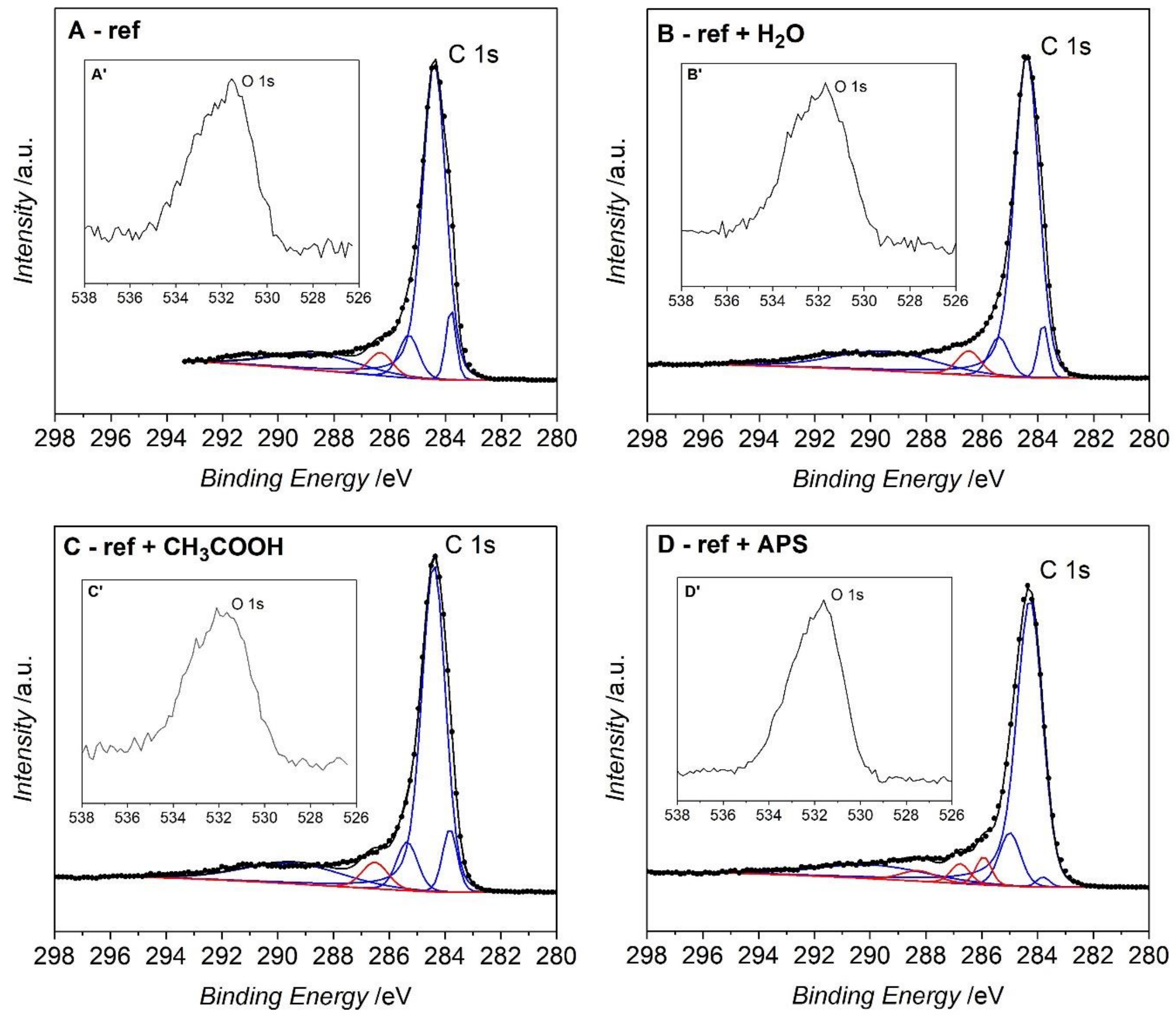

Figure S3. XPS O 1s and C 1s spectra and deconvolution of reference graphene nanoplatelets where A- unmodified material and treated by B- H_2O, C- CH_3COOH, D- APS.

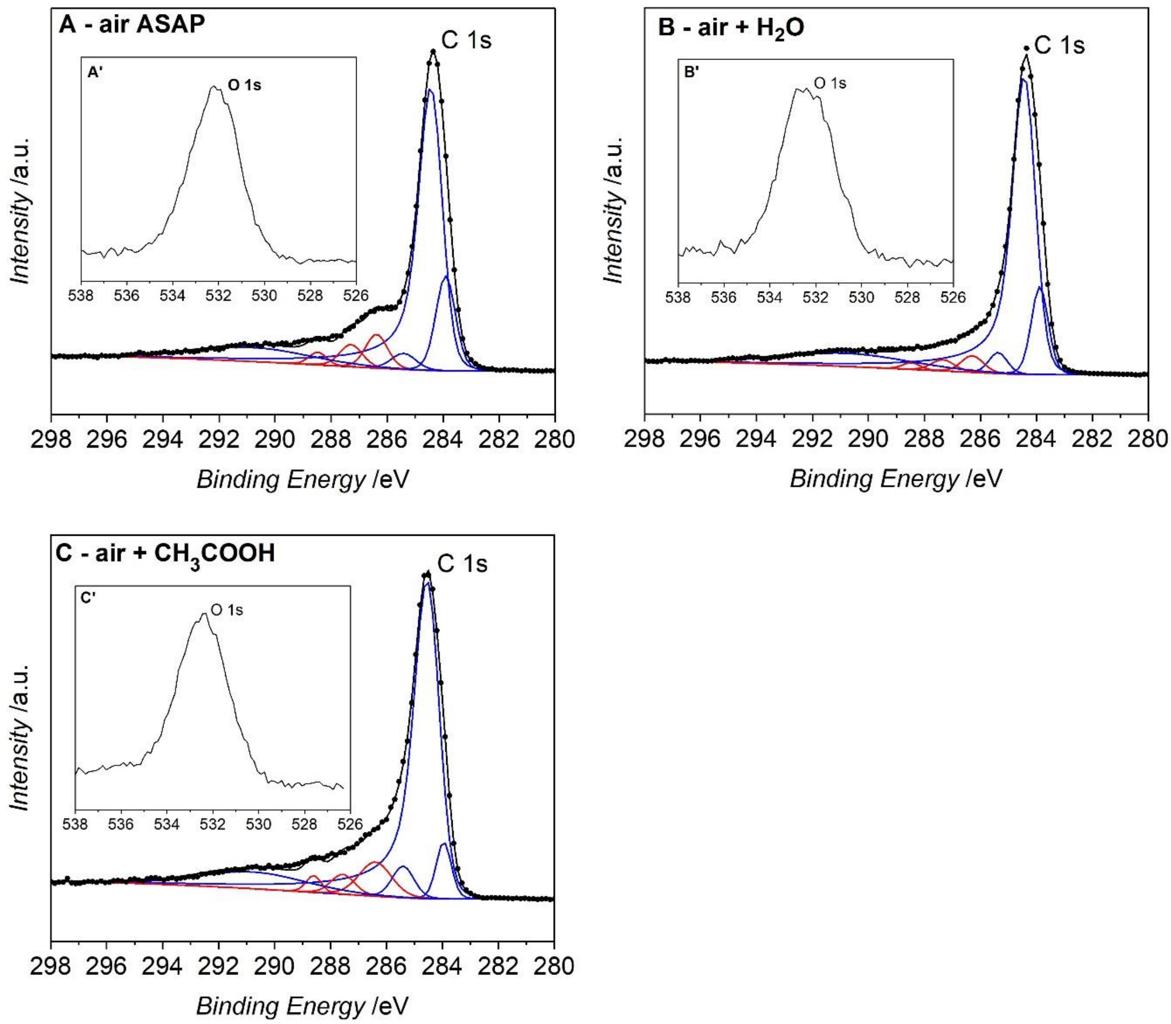

Figure S4. XPS O 1s and C 1s spectra and deconvolution of graphene nanoplatelets treated by air plasma condition 5 min, 0.7 mbar to optimise towards maximum work function where A- just after plasma treatment, B- after immersion in H_2O, C- after immersion in CH_3COOH.

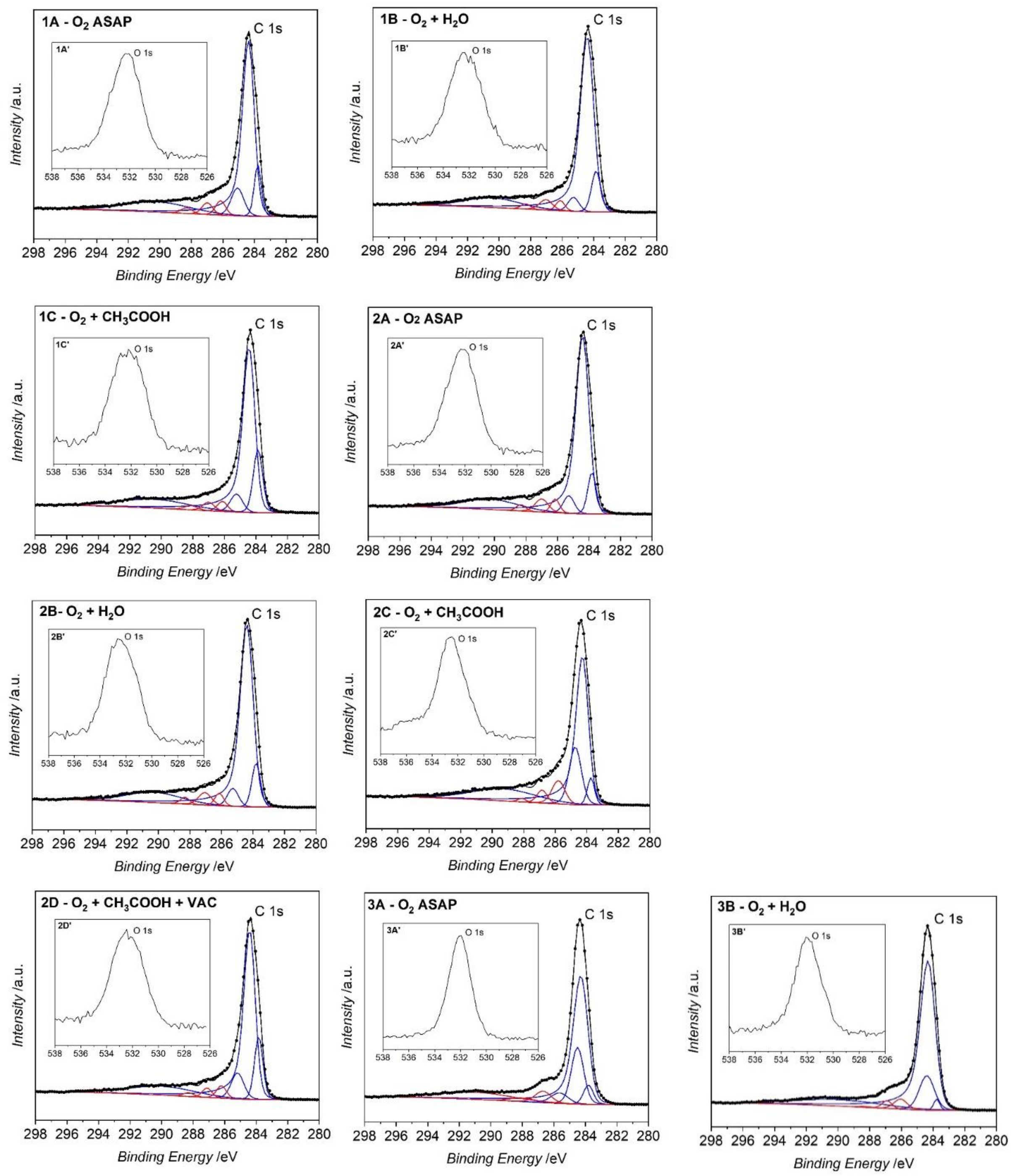

Figure S5. XPS O 1s and C 1s spectra and deconvolution of graphene nanoplatelets treated by oxygen plasma under different conditions to optimise towards maximum work function. Samples marked as 1 were treated during 1 min and 0.2 mbar pressure, 2 for 5 min, 0.7 mbar pressure, 3 for 5 min, 0.2 mbar pressure where A- just after plasma treatment, B- after immersion in H_2O, C- after immersion in CH_3COOH, D- after immersion in CH_3COOH and dried in vacuum.

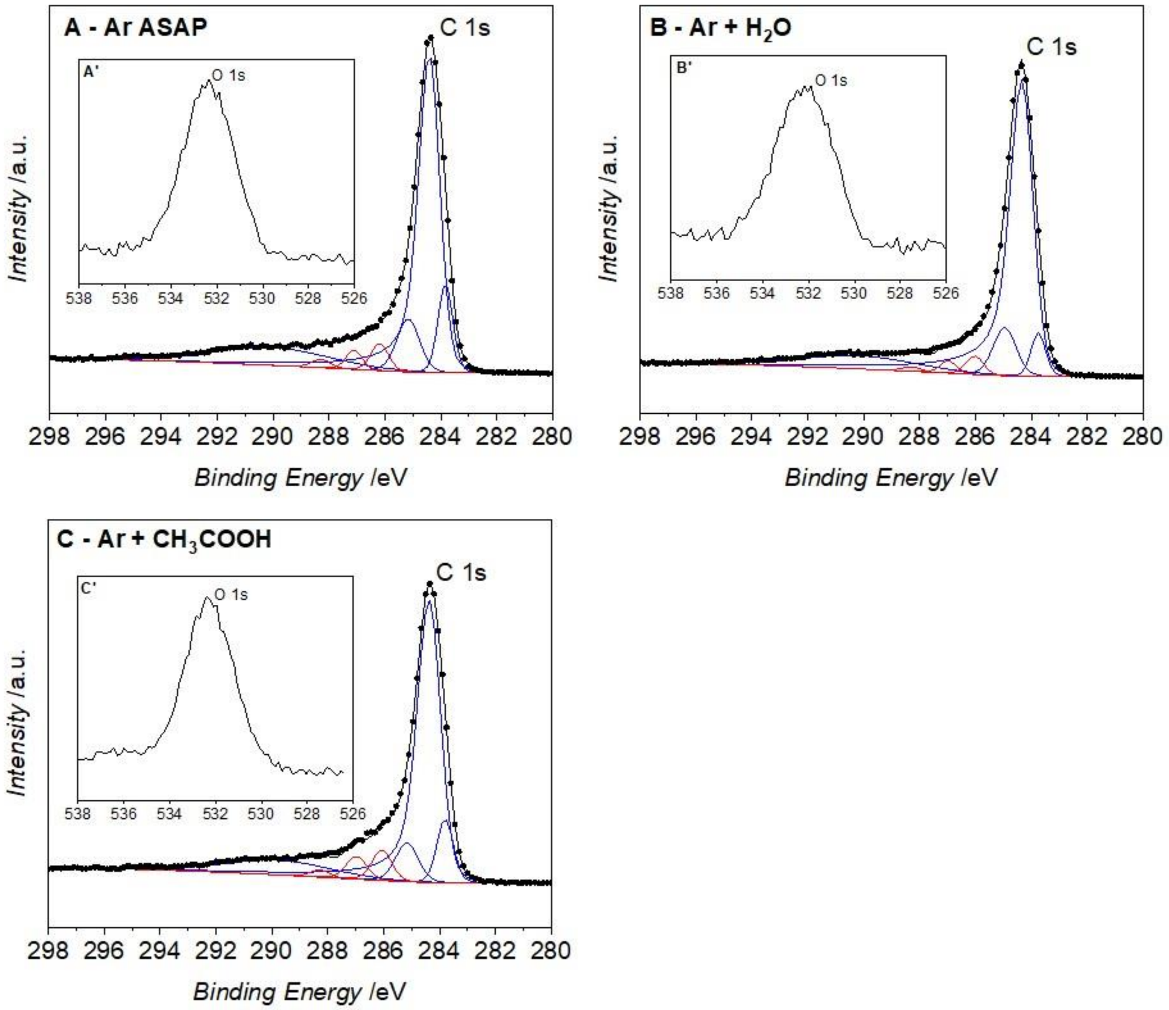

Figure S6. XPS O 1s and C 1s spectra and deconvolution of graphene nanoplatelets treated by argon plasma condition 5 min, 0.7 mbar to optimise towards maximum work function where A- just after plasma treatment, B- after immersion in H_2O, C- after immersion in CH_3COOH.

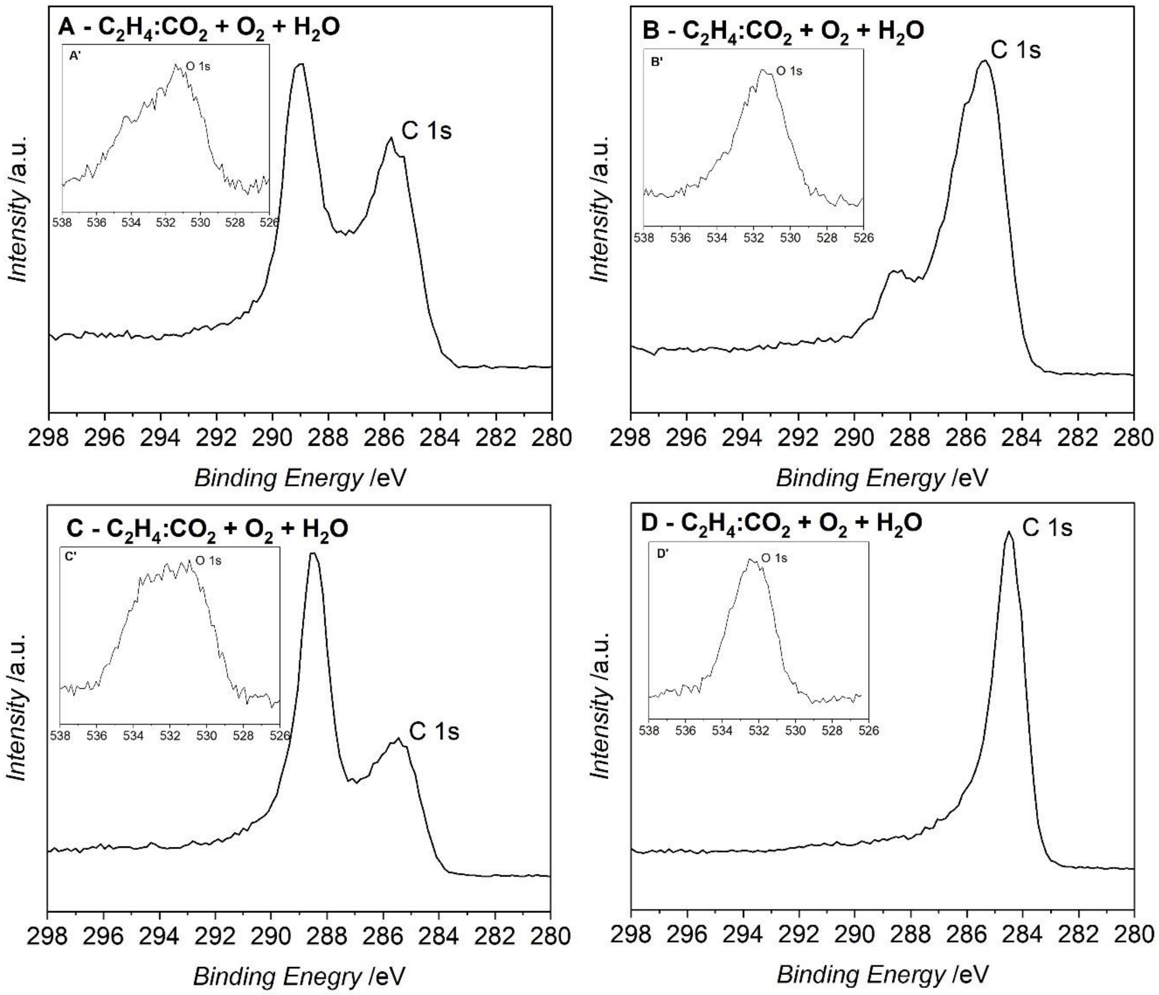

Figure S7. XPS O 1s and C 1s spectra of graphene nanoplatelets modified under plasma conditions 5 min, 0.8 mbar C_2H_4:CO_2 and optimisation of time oxygen plasma treatment during oxidising process under 0.2 mbar pressure for A – 6 s, B – 30 s, C – 1 min, D – 5 min.

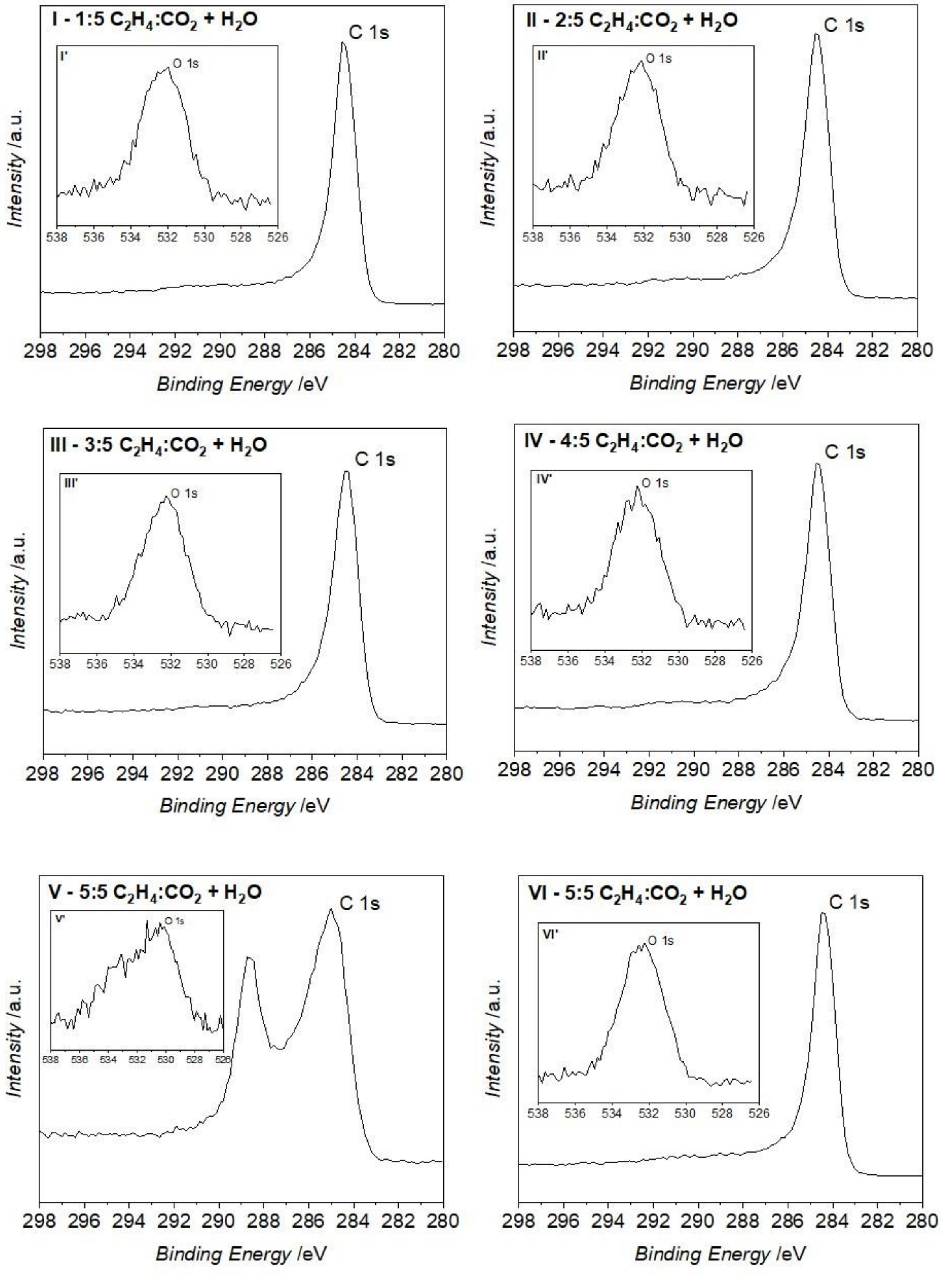

Figure S8. XPS O 1s and C 1s spectra of graphene nanoplatelets and optimisation of the ratio in the mixture of C_2H_4:CO_2 for I - V under plasma conditions 5 min, 0.8 mbar and sample VI treated by 5:5 C_2H_4:CO_2 under 5 min and 0.2 mbar pressure.

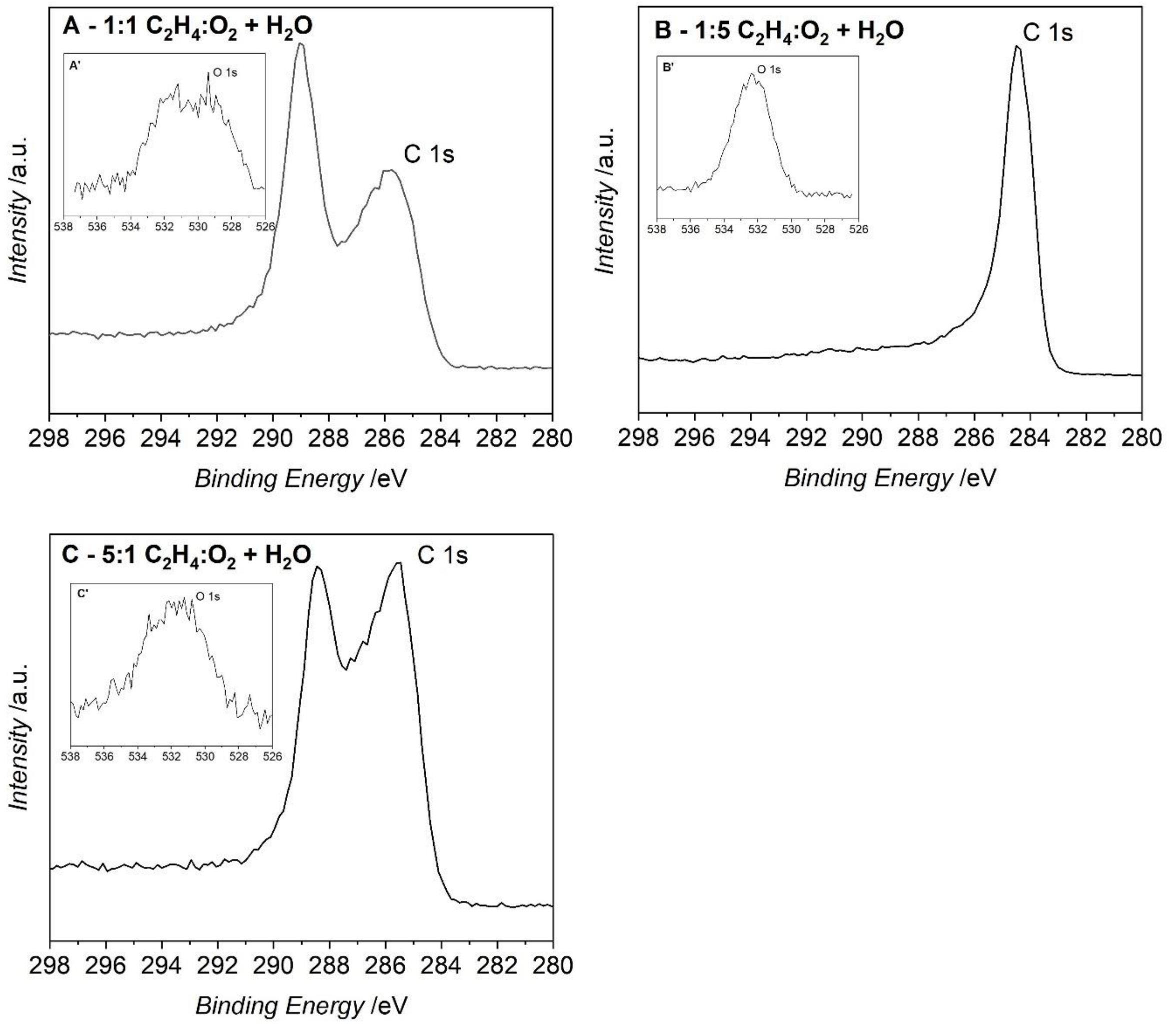

Figure S9. XPS O 1s and C 1s spectra of graphene nanoplatelets modified by the mixture C_2H_4:O_2 under plasma conditions 5 min, 0.8 mbar at varied ratios: A) 1:1, B) 1:5, and C) 5:1.
